# Widening gap in life expectancy between patients with heart failure living in most and least deprived areas: a longitudinal cohort study

**DOI:** 10.1186/s12916-025-04137-4

**Published:** 2025-05-28

**Authors:** O. I. Brown, M. Drozd, H. MacGowan, M. McGinlay, R. Burgess, S. Straw, A. D. Simms, V. K. Gatenby, A. Sengupta, A. M. N. Walker, C. Saunderson, M. F. Paton, K. I. Bridge, J. Gierula, K. K. Witte, R. M. Cubbon, M. T. Kearney

**Affiliations:** 1 Leeds Institute of Cardiovascular and Metabolic Medicine, University of Leeds, Leeds, UK; 2 Department of Cardiology, Leeds Teaching Hospitals NHS Trust, Leeds, UK

**Keywords:** Heart failure, Socioeconomic deprivation, Actuarial survival, Health inequality

## Abstract

**Background:**

Socioeconomic deprivation is associated with adverse clinical outcomes in patients with heart failure (HF). However, in the context of improved medical and device therapy for HF, it is unknown whether the influence of socioeconomic deprivation on HF outcomes is changing over time, especially in relation to evolving life expectancy patterns in the general population. Therefore, we aimed to describe temporal trends in the association of socioeconomic deprivation with loss of actuarially predicted life expectancy amongst ambulatory patients with HF.

**Methods:**

Between 2006 and 2014, 1802 patients (73.2% male, mean age 69.6 years) with HF and left ventricular ejection fraction ≤ 45% were consecutively recruited across four hospitals in the United Kingdom (UK). Patients were stratified into socioeconomic deprivation tertiles defined by the UK Index of Multiple Deprivation (IMD) score with IMD tertile 1 denoting the least deprived and IMD tertile 3 the most deprived. The primary outcome was all-cause mortality, and relative survival predictions—in relation to age- and sex-matched background mortality rates—were calculated using UK National Life Tables. Relative survival was illustrated in terms of excess mortality risk and years of life expectancy lost. Recruitment period was split into 3-year intervals (2006–2008, 2009–2011 and 2012–2014).

**Results:**

During a median follow-up of 5.0 years, 1302 participants (72.3%) died. Unadjusted mortality rate was highest in tertile 2. However, adjusted to the age–sex matched UK population, a stepwise increase in excess mortality risk was observed across tertiles, with tertile 1 experiencing an excess mortality risk of 11.1% (95% CI: 6.1–16.1%) and tertile 3 24.2% (95% CI: 19.4–28.0%). This corresponded to a loss of life expectancy of 1.76 years (95% CI: 1.50–2.03) for tertile 1 and 2.30 years (95% CI: 2.03–2.57) for tertile 3 over a 10-year period. We observed disparity in actuarial survival between tertiles over time, with participants in tertile 1 losing less life expectancy at 10 years compared to those in tertiles 2 and 3. However this was only statistically significant for those recruited between 2012 and 2014 (*p* < 0.05).

**Conclusions:**

The impact of socioeconomic deprivation on HF outcomes in an unselected diverse UK population appears to have worsened over time.

**Supplementary Information:**

The online version contains supplementary material available at 10.1186/s12916-025-04137-4.

## Background

Chronic heart failure (HF) is a clinical syndrome defined by dyspnoea, venous congestion and elevated natriuretic peptides, leading to increased hospitalisation and premature mortality [1]. Whilst the standardised incidence of HF is declining, the prevalence and burden of HF in the United Kingdom (UK), both to individuals and society, continues to grow. This is driven by an ageing population and improved survival following the widespread adoption of comprehensive medical and device therapy for heart failure with reduced ejection fraction (HFrEF) [2, 3]. Despite this, there remain inequalities in access to services and therapies for people with HF within the UK [4].

Socioeconomic deprivation is associated with higher mortality and increased rates of hospital admissions in HF patients. Moreover, patients facing socioeconomic deprivation often encounter multilevel barriers to healthcare contributing to healthcare inequalities [5–7]. At the systems level, healthcare facilities serving low-income populations frequently lack adequate resources, particularly regarding access to primary care and specialty services in underserved areas. This may delay diagnosis and treatment initiation, leading to more advanced disease progression at the time of presentation [8, 9]. Whilst at a provider level, healthcare worker implicit biases, stereotyping and communication barriers can influence how patients are perceived and treated, potentially resulting in unequal care [10].

Socioeconomic deprivation is linked to higher rates of comorbidities, such as diabetes, hypertension and obesity, which complicate the management of HF and contribute to poorer outcomes [2, 11]. Furthermore, patients in lower socioeconomic groups may face barriers to adhering to prescribed therapies, such as medication costs or limited health literacy, reducing the effectiveness of evidence-based treatments [12]. Psychosocial stress, poor nutrition and limited social support networks further exacerbate the burden of HF in deprived populations, contributing to the observed disparities in outcomes [12].

However, the effect of socioeconomic deprivation on HF mortality over time remains unknown and challenging to assess given changing rates in comorbidities and an ageing population. Moreover, previous studies have demonstrated marked discrepancy between patient-predicted survival and survival estimates from prognostic models, highlighting the importance of improving communication around this critical and sensitive issue [13].

Given this, we aimed to compare the observed survival of individuals with HF to that of an age- and sex-matched actuarial control population, and to assess the association of socioeconomic deprivation with this over time.

## Methods

The design of the United Kingdom Heart Failure Evaluation and Assessment of Risk Trial (UK-HEART-2), a prospective cohort study of ambulatory heart failure patients within the UK, has been previously published [14, 15]. The overarching aim of UK-HEART-2 was to identify prognostic markers in patients with HF and reduced left ventricular ejection fraction (LVEF), receiving contemporary evidence-based therapy.

Between July 2006 and December 2014, consecutive patients with stable symptoms and/or signs of HF for at least 3 months and an LVEF ≤ 45% under the care of four specialist heart failure outpatient clinics within West Yorkshire in the UK were enrolled. The secondary care hospital where patients were reviewed was determined by their residential postcode, which was also used to calculate their Index of Multiple Deprivation (IMD) score. All patients provided informed written consent and the study was conducted in accordance with the principles outlined in the Declaration of Helsinki. Ethical approval of the study was granted by the Leeds West Research Ethics Committee prior to study commencement.

All patients underwent transthoracic echocardiography, resting 12-lead ECGs and blood testing for measurement of full blood count, electrolytes and serum creatinine. Functional status was assessed using the New York Heart Association (NYHA) classification. Total daily doses of angiotensin converting enzymes inhibitors, angiotensin receptor blockers, beta-blockers and loop diuretic were collected at recruitment; these were normalised to maximum licenced HF dose as previously described [16]. Receipt of cardiac resynchronisation therapy or implantable cardioverter defibrillator was assessed during the 6-month period after recruitment.

For our secondary analysis looking at the association of socioeconomic deprivation with mortality over time, we split recruitment period into three yearly intervals (2006 to 2008, 2009 to 2011 and 2012 to 2014).

### Socioeconomic status

Socioeconomic status was determined using individual patient postal codes, which were mapped to one of 32,482 geographical regions, each representing approximately 1500 people. These regions were defined according to the IMD which measures local socioeconomic deprivation. Since our cohort recruitment spanned three official IMD updates (2007, 2010 and 2015), we assigned the IMD rank/score based on the update closest to each patient’s recruitment date. IMD provides a current index of socioeconomic deprivation, compiled from data collected by various UK government and non-government agencies, and is recognised as a valid indicator of overall deprivation at the geographical level. It generates a composite deprivation score for each region, weighted by domains such as income (22.5%), employment (22.5%), health and disability (13.5%), education, skills and training (13.5%), barriers to housing and services (9.3%), crime (9.3%) and living environment (9.3%). In our analysis, participants were divided into tertiles with tertile 1 denoting the least deprived and tertile 3 the most deprived.

### Outcome assessment

The primary outcome measure was all-cause mortality at 10 years. Vital status data were collected using linked national electronic records from the Office of National Statistics. Final censorship occurred on the 23rd of October 2024. For our sensitivity analysis looking at the associations with deprivation by period of enrolment, duration of follow-up was limited to 10 years to ensure equal follow-up durations.

Actuarial survival predictions were derived from the UK National Life Tables (UK-NLT), an official survival estimation measure produced by the Human Mortality database [17]. The UK-NLT provide annual death rates by sex and age for any given year. This provides the baseline survival for members of the public with this age and sex, which we used as a reference control population.

### Statistical analysis

Statistical analysis was performed using RStudio version 4.1.1. Analysis used the R suite ‘tidyverse’, whilst plots were compiled using the embedded ‘ggplot2’ package. Relative survival analysis was performed using the ‘Survival’ and ‘Relsurv’ packages (https://www.jstatsoft.org/article/view/v087i08).

Patient characteristics are reported using the mean and standard deviation for continuous variables, with categorical variables summarised using the count of each class and the percentage of the dataset it represents. Unadjusted cumulative mortality rates describing the observed cohort survival were calculated and stratified by deprivation tertile. Relative survival data were illustrated with excess mortality rate (defined as the observed mortality rate minus the expected age–sex matched mortality rate in the general population defined by the UK-NLT) and by calculating excess loss of life expectancy stratified by deprivation tertile, and according to period of recruitment. Recruitment period was split into periods of three consecutive calendar years. Wald confidence intervals (CIs) are used for mortality rate, whilst 500 bootstrap samples are used to produce CIs for years of life lost, with ANOVA used to compare the mean years of life lost between periods of recruitment. Missing data were not imputed.

## Results

We recruited 1802 patients who had a mean age of 69.6 years (SD 12.5) and 1319 (73.2%) were male. Descriptive data comparing demographics, comorbidities, symptom severity and HF management by socioeconomic deprivation tertiles are shown in Table 1. Patients from areas of higher socioeconomic deprivation were more likely to be younger, male, have greater body mass index (BMI) be of black, Asian or minority ethnicity and have chronic obstructive pulmonary disease. Moreover, they had greater symptom burden and were less likely to receive cardiac implantable electronic device therapy.

**Table 1:** Baseline characteristics by socioeconomic deprivation tertile

	Total population (*n* = 1802)	Tertile 1 (*n* = 600)	Tertile 2 (*n* = 601)	Tertile 3 (*n* = 601)	*p* value
Age, years	69.6 (12.5)	71.3 (11.5)	70.5 (12.2)	67.0 (13.3)	0.002
Male, y/n	1319 (73.2)	468 (78.0)	437 (72.7)	414 (68.9)	0.005
IMD score	26.6 (18.6)	8.8 (3.1)	21.4 (5.7)	49.6 (10.9)	< 0.001
BMI, kg/m^2^	28.1 (6.0)	27.2 (4.9)	28.5 (5.4)	28.6 (7.6)	< 0.001
BAME, y/n	71 (3.9)	22 (3.7)	16 (2.7)	33 (5.4)	0.037
Ischaemic aetiology, y/n	1067 (59.2)	366 (61.0)	356 (59.2)	345 (57.4)	0.547
Diabetes, y/n	504 (28.0)	151 (25.2)	166 (27.6)	187 (31.1)	0.070
COPD, y/n	284 (15.8)	72 (12.0)	99 (16.4)	113 (18.8)	0.003
CKD 4 or above, y/n	141 (7.8)	44 (7.3)	43 (7.2)	54 (9.0)	0.422
NYHA class 3/4, y/n	555 (30.8)	161 (26.6)	183 (30.7)	211 (35.2)	0.005
LVEF, %	32 (9)	31 (9)	32 (10)	32 (10)	0.468
QRS, ms	123 (31)	127 (31)	125 (31)	118 (29)	0.090
SBP, mmHg	122 (22)	121 (21)	123 (21)	123 (23)	0.261
DBP, mmHg	71 (11)	71 (11)	71 (11)	72 (12)	0.186
HR, bpm	75 (16)	74 (15)	75 (16)	76 (16)	0.748
Betablocker, y/n	1523 (84.7)	529 (87.4)	488 (82.2)	506 (84.5)	0.039
Bisoprolol equivalent dose, mg	3.9 (3.4)	4.1 (3.4)	3.7 (3.2)	3.8 (3.4)	0.260
ACEi/ARB y/n	1626 (90.4)	558 (92.2)	524 (88.2)	544 (90.8)	0.057
Ramipril equivalent dose, mg	4.9 (3.5)	5.1 (3.5)	4.8 (3.6)	4.9 (3.5)	0.910
Loop diuretic y/n	1340 (74.6)	459 (75.9)	444 (74.4)	442 (74)	0.669
Furosemide equivalent dose, mg	51 (50)	52 (50)	49 (46)	52 (51)	0.644
MRA y/n	689 (38.3)	235 (38.8)	219 (36.9)	235 (39.2)	0.667
CRT y/n	455 (25.3)	181 (29.9)	154 (25.8)	120 (20)	< 0.001
ICD y/n	210 (11.7)	95 (15.7)	65 (10.9)	50 (8.3)	< 0.001

Continuous data is presented as mean and standard deviation (SD). Categorical data is *n* (%). *p* value for continuous variables from ANOVA and categorical variables from chi^2^ testing respectively

*Abbreviations* *ACEi* angiotensin converting enzyme inhibitor, *ARB* angiotensin receptor blocker, *BAME* Black, Asian and Minority Ethnic, *BPM* beats per minute, *BMI* body mass index, *CRT* cardiac resynchronisation therapy, *CKD* chronic kidney disease, *COPD* chronic obstructive pulmonary disease, *DBP* diastolic blood pressure, *HR* heart rate, *ICD* implantable cardiac defibrillator, *IMD* Index of Multiple Deprivation, *LVEF* left ventricular ejection fraction, *MRA* mineralocorticoid receptor antagonist, *mg* milligram, *NYHA* New York Heart Failure Association, *SBP* systolic blood pressure

Across differing recruitment periods from earliest to latest, patients were broadly older at presentation, less likely to have HF due to an ischaemic aetiology, had greater beta-blocker use at higher doses and had a lower burden of symptoms irrespective of socioeconomic status (Additional file 1: Tables S1–S3). Use of cardiac resynchronisation therapy (CRT) and implantable cardiac defibrillators (ICD) decreased over time in all groups. Mineralocorticoid receptor antagonist use significantly decreased over time only in patients from areas with higher socioeconomic deprivation (Additional file 1: Table S3).

### Association with outcome

**Fig. 1:**
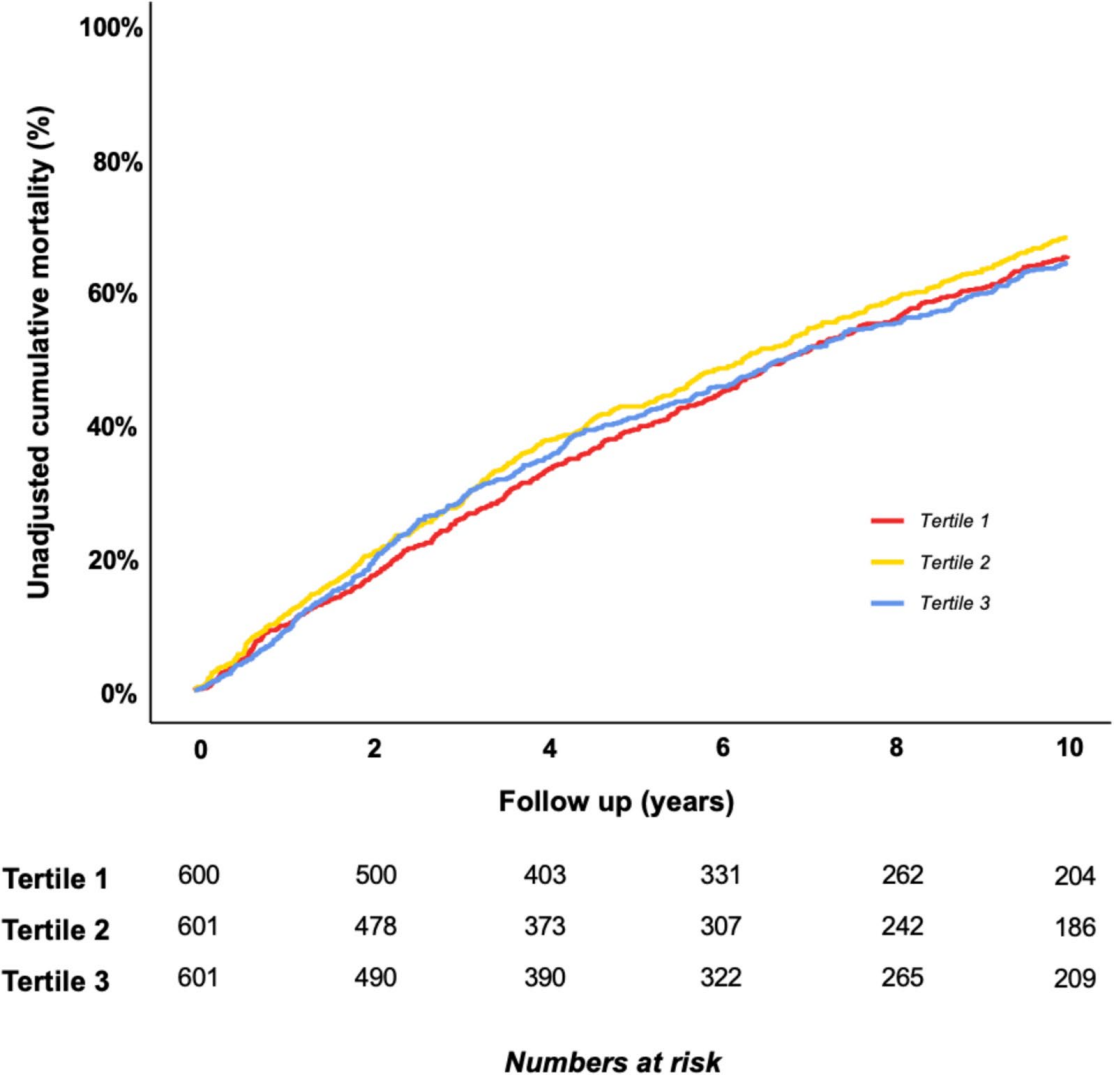
Unadjusted cumulative mortality over ten years by socioeconomic deprivation tertile of study cohort

**Fig. 2:**
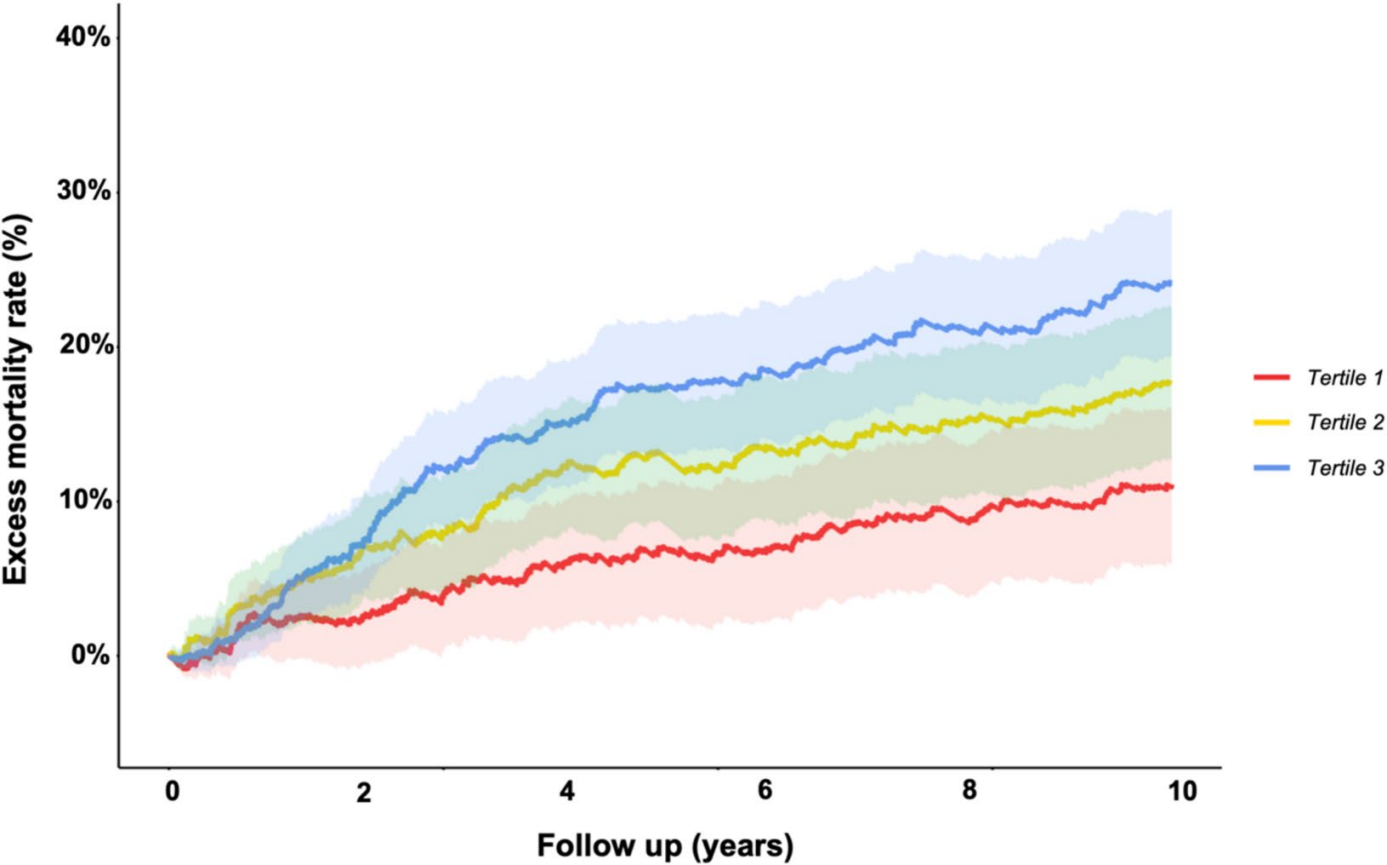
Excess mortality over ten years by socioeconomic deprivation tertile in the study cohort Excess mortality rate is expressed as percentage with shaded areas representing 95% confidence intervals

During a median follow-up of 5.0 years (25th to 75th centile: 2.7–7.5), a total of 1302 participants (72.3%) died. The unadjusted cumulative mortality rates were similar across tertiles of socioeconomic deprivation; however, by 10 years of follow-up, mortality was nominally highest in tertile 2 (Fig. 1). To quantify differences in excess mortality risk by socioeconomic deprivation, we constructed relative survival models. We observed a stepwise increase in excess mortality risk across the socioeconomic deprivation tertiles, with participants in tertile 3 (representing the most socioeconomic deprivation) having the greatest excess mortality risk (Fig. 2 and Table 2). After 10 years, the excess mortality risk for participants in IMD tertile 3 relative to the general population was 24.2% (95% CI: 19.4–28.9%), compared to 11.1% (95% CI: 6.1–16.1%) for IMD tertile 1 (Table 2).

**Table 2:** Relative survival by socioeconomic deprivation tertile

	Excess mortality risk during specified period, % (95% CI)		
	Three years	Five years	Ten years
Tertile 1	4.9% (95% CI: 1.3–8.7)	6.9% (95% CI: 2.4–11.3)	11.1% (95% CI: 6.1–16.1)
Tertile 2	8.1% (95% CI: 4.3–11.9)	12.6% (95% CI: 8.1–17.1)	17.6% (95% CI: 12.7–22.6)
Tertile 3	12.6% (95% CI: 8.8–16.3)	17.5% (95% CI: 13.1–21.7)	24.2% (95% CI: 19.4–28.9)
	Years of life lost during specified period, years (95% CI)		
	Three years	Five years	Ten years
Tertile 1	0.22 (95% CI: 0.15–0.28)	0.56 (95% CI: 0.44–0.69)	1.76 (95% CI: 1.50–2.03)
Tertile 2	0.29 (95% CI: 0.22–0.36)	0.73 (95% CI: 0.60–0.86)	2.14 (95% CI: 1.86–2.41)
Tertile 3	0.29 (95% CI: 0.23–0.36)	0.77 (95% CI: 0.65–0.90)	2.30 (95% CI: 2.03–2.57)

Relative survival is expressed as % excess mortality risk and years of life expectancy lost relative to age- and sex-matched UK population with 95% confidence intervals (CI)

**Fig. 3:**
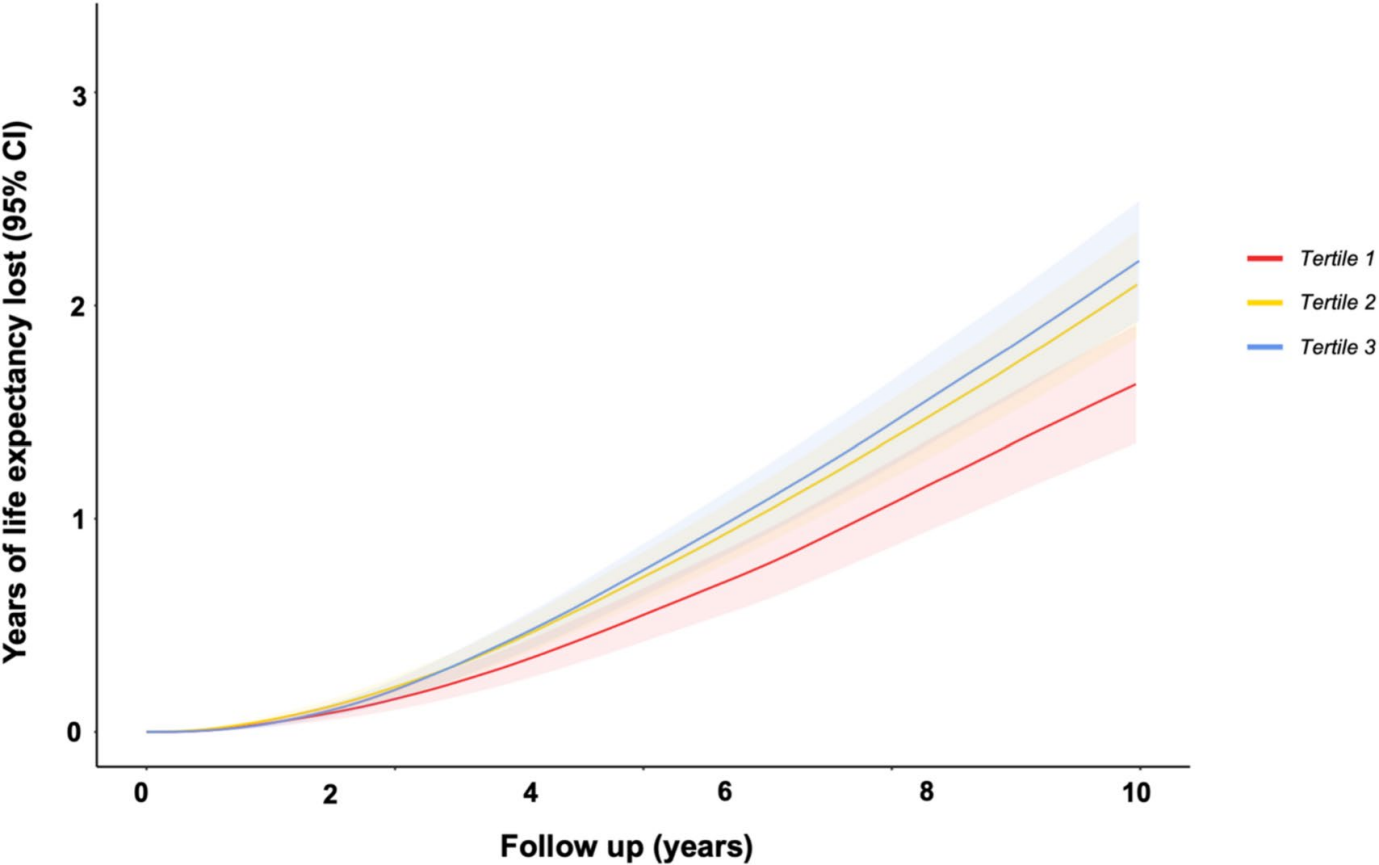
Years of life expectancy lost over ten years by socioeconomic deprivation tertile Life expectancy loss is expressed in years, with shaded areas representing 95% confidence intervals

**Fig. 4:**
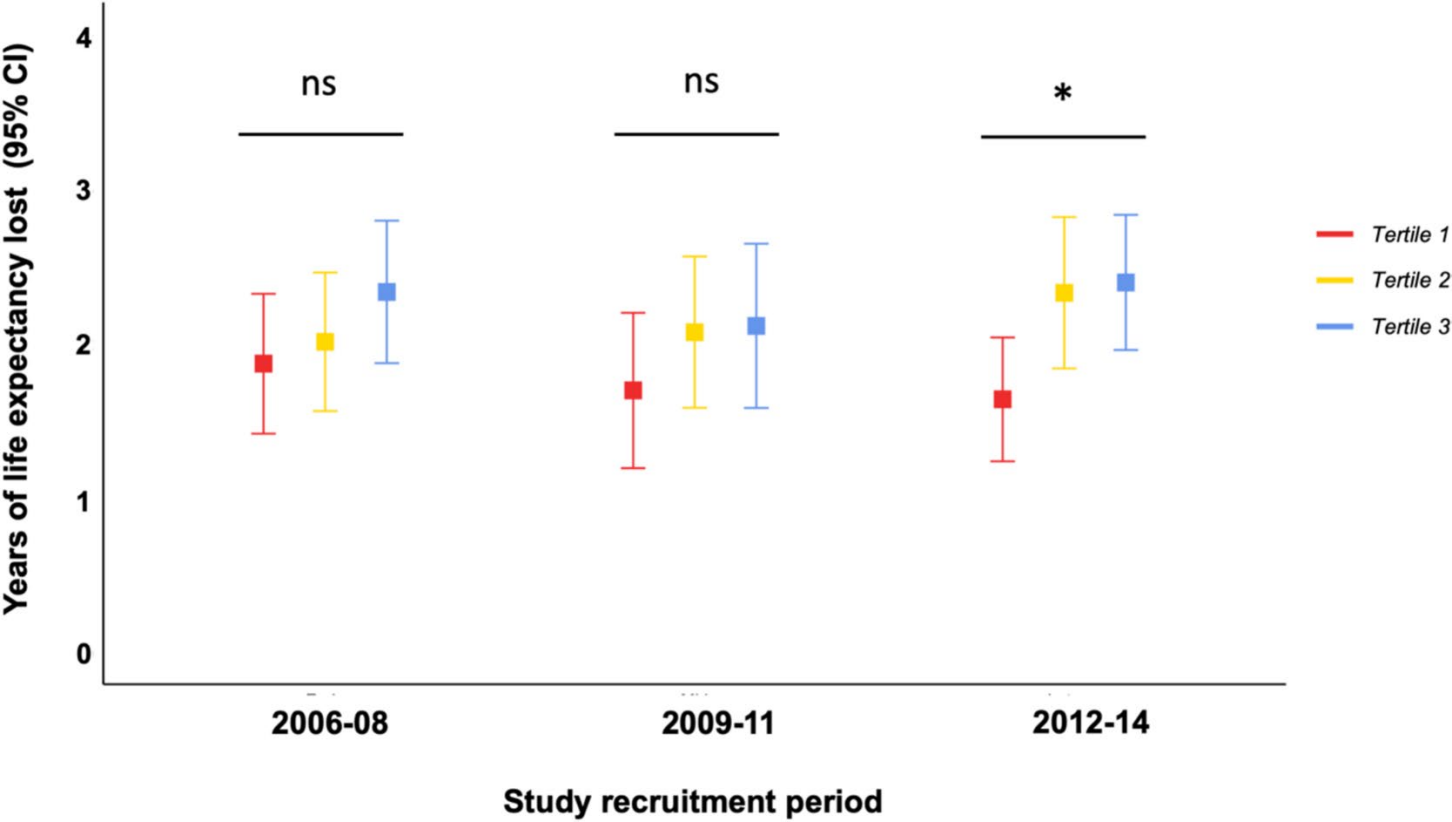
Years of life expectancy lost at 10-year follow up by socioeconomic deprivation tertile and study recruitment period Life expectancy loss is expressed in years, with errors bars as 95% confidence intervals. Statistical significance was assessed using ANOVA across study recruitment periods. Abbreviations: not significant (ns), * *p* < 0.05

In terms of years of life lost, participants in tertile 1 (lowest deprivation) experienced a cumulative loss of 1.76 years (95% CI: 1.50–2.03), whilst those in IMD tertile 3 experienced the highest cumulative loss, with 2.30 years (95% CI: 2.03–2.57), a difference of 6.5 months over a 10-year period (Fig. 3, Table 2). Moreover, whilst there was no statistically significant difference in life lost over a 10-year period by IMD tertile in the first and second recruitment periods, during final recruitment period participants in IMD tertile 1 lost significantly less life at 10 years compared to those in IMD tertiles 2 and 3 (Fig. 4).

## Discussion

We present a comprehensive analysis of the effects of socioeconomic deprivation on survival in relation to actuarial predictions in a large, unselected cohort of patients with HF. By considering actuarial estimates of survival, we have shown a direct relation between socioeconomic deprivation and all-cause mortality, with patients from areas of high socioeconomic deprivation losing 2.30 years of life compared to the age- and sex-matched general population over a 10-year period. Moreover, there appears to be a widening disparity in the effects of socioeconomic deprivation on survival in relation to actuarial predictions over time, with patients with HF from the least deprived areas losing significantly less life over the recruitment period compared to those from areas with higher socioeconomic deprivation.

We have previously shown that patients with HF have a 2.4-fold excess loss of life compared to an age- and sex-matched general population [14]. Moreover, prior studies have demonstrated an inverse relation between socioeconomic deprivation and disease-free survival in patients with HF [18–20], a phenomenon driven by increased risk of non-cardiovascular mortality and hospitalisation [5]. No study has quantified the effects of socioeconomic deprivation on survival in relation to actuarial predictions amongst patients with HF.

Multimorbidity amongst patients with HF is common and more prevalent in patients from areas of higher socioeconomic deprivation [21, 22] and may account for part of the adverse association between socioeconomic deprivation and mortality [23]. Comorbidity adds complexity to the management of HF, as it can make diagnosis more difficult, prescribing guideline-directed medical therapy more challenging and augurs a worse prognosis [24]. Comorbidities more frequently accrue at a younger age in patients with HF from areas of higher socioeconomic deprivation [2], which we also observed in our study with patients in the most deprived group were younger and had the greatest prevalence of chronic obstructive pulmonary disease and diabetes. Younger patients have the largest potential number of years of life expectancy to lose and the greatest potential for life expectancy gained with effective medical therapy [25], and this may explain the differences in survival seen in our cohort. In addition, patients from the most deprived areas in our study were least likely to receive device therapy and were the only group to see a significant reduction in the use of mineralocorticoid receptor antagonists over the study period; both findings are in keeping with observational data from the USA [26, 27].

We observed an apparent worsening of disparity in actuarial survival by socioeconomic status over time, with only patients from areas with lower socioeconomic deprivation seeing a reduction in loss of years of life expectancy across recruitment period. In the only other study reporting the temporal effects of socioeconomic status on heart failure mortality, Taylor et al. demonstrated in a cohort of 55,959 primary care patients with a new diagnosis of HF that whilst there was little difference between 1- and 5-year survival in the most deprived and least deprived groups over the study period, 10-year survival was significantly lower in the most deprived group [3]. However, this study did not formally classify HF based on systolic function and the HF diagnosis was derived from GP coding. In addition, it only included patients in primary care who may have a different prognosis to those being managed in the hospital setting.

The reasons behind this widening disparity are not clear. The present study was conducted during a period where there was significant change within UK health care policy, amongst a wider context of economic austerity [28]. Between 2009 and 2011, public expenditure within the UK was cut by 2.2% [29]. Although expenditure on overall healthcare was protected, social care, local government and income support were not protected and cuts to these areas have direct and indirect effects on health, including increasing unemployment, poverty and homelessness [29, 30]. These policies disproportionately impact people of lower socioeconomic status [28], and during this period of austerity, all-cause mortality rates increased across the UK, particularly amongst those in the UK from areas of higher socioeconomic deprivation [31]. Our results reflect this pattern and austerity may in part be responsible for this widening disparity.

Tackling the impact of socioeconomic deprivation on health is complex and requires a multilevel approach [7]. At the system level, increasing funding and resource allocation to facilities serving socioeconomically deprived populations is critical to address disparities in access to care [32]. Additionally, educating healthcare providers on the social determinants of health can help them better understand the context of their patients’ lives [33]. Encouraging shared decision-making practices enables patients, regardless of their health literacy levels, to actively participate in their care, fostering greater equity in outcomes [34].

Patients from areas of higher socioeconomic deprivation are historically underrepresented in HF randomised controlled trials and analyses by socioeconomic status are not routinely reported [35]. Although there is no biological reason to believe the effect of HF therapies varies across socioeconomic strata, other factors such as medical compliance and healthcare access are influenced by socioeconomic status and could potentially interact with treatment efficacy [35]. To address this, initiatives should be established to widen and promote trial recruitment of people from areas of lower socioeconomic status and reporting of treatment effects according to socioeconomic status should be mandatory.

Effective communication with patients and relatives regarding disease prognosis is reliant on the application and understanding of survival statistics [36]. More widely used measures of survival including hazard ratio, relative and absolute risk do not account for the expected rate of death of the general population without disease, nor reflect the reality that life is finite. In addition, patients with HF frequently overestimate their life expectancy compared to model-based predictions, particularly those patients with more severe disease [13], whilst repeated and prolonged hospital admission is common amongst patients with HF towards the last year of life [37]. Therefore, the ability to provide more nuanced and understandable illustrations of disease prognosis to patients with HF using relative survival measures may help facilitate advanced care planning discussions and ensure patients have realistic expectations of their disease prognosis [12].

### Strengths and limitations

First, IMD is a measure of area-based deprivation and does not reflect the deprivation of an individual, so some patients’ personal socioeconomic deprivation may have been over or underestimated. Second, participants were recruited before the widespread use of other disease modifying therapies for HF including angiotensin receptor/neprilysin inhibitors and sodium glucose co-transporter inhibitors which may improve cardiovascular outcomes. Therefore, our survival estimates may be lower than contemporary estimates [38, 39]. In addition, our estimates do not reflect or account for quality of life, merely the number of years of life expectancy lost. Our analysis stratifies participants and inherently accounts for two strong predictors of mortality, age and biological sex, using population life tables. But we did not adjust for other covariates such as medication use, ethnicity, comorbidities or physical characteristics. However, these features are key components of socioeconomic status and reflect important mechanisms through which socioeconomic status affects health outcomes. Adjusting for these factors could obscure their contribution to the disparities observed between socioeconomic strata, as they are intrinsic to the effects of deprivation. Finally, our study used expected survival data from the UK and our estimates may not be generalisable to the rest of the world.

## Conclusions

We have shown that rising socioeconomic deprivation is associated with incremental loss of life expectancy amongst patients with HF. In addition, there is evidence of widening disparity in the amount of life expectancy lost between socioeconomic groups. Our data provides understandable and quantifiable estimates of loss of life expectancy for patients with HF and may help facilitate more nuanced conversations regarding advanced care planning. Future research should explore whether targeted interventions in health, social and economic policy can reduce the socioeconomic disparities in survival observed amongst patients with HF.

## Supplementary Information

Additional file 1: Tables S1–S3 contains tables demonstrating clinical characteristics of socioeconomic deprivation tertiles over study period. Table S1 Characteristics of socioeconomic deprivation tertile 1 over study period. Table S2 Characteristics of socioeconomic deprivation tertile 2 over study period. Table S3 Characteristics of socioeconomic deprivation tertile 3 over study period.

## Data Availability

No datasets were generated or analysed during the current study.

## Abbreviations

ACEI: Angiotensin converting enzyme inhibitor
ARB: Angiotensin receptor blocker
BAME: Black, Asian and Minority Ethnic
BMI: Body mass index
BPM: Beats per minute
CI: Confidence interval
CKD: Chronic kidney disease
COPD: Chronic obstructive pulmonary disease
CRT: Cardiac resynchronisation therapy
DBP: Diastolic blood pressure
HF: Heart failure
HFrEF: Heart failure with reduced ejection fraction
HR: Heart rate
ICD: Implantable cardiac defibrillator
IMD: Index of Multiple Deprivation
LVEF: Left ventricular ejection fraction
MRA: Mineralocorticoid receptor antagonist
NYHA: New York Heart Association
SBP: Systolic blood pressure
SD: Standard deviation
UK: United Kingdom
UKH2: United Kingdom Heart Failure Evaluation and Assessment of Risk Trial
UK-NLT2: UK National Life Tables
