# Supplementary material for: Widening gap in life expectancy between patients with heart failure living in most and least deprived areas: a longitudinal cohort study
Source: BMC Med. 2025 May 28;23:303. doi: 10.1186/s12916-025-04137-4 (PMC12121114; doi:10.1186/s12916-025-04137-4)
Supplement: Supplementary file 1 — Additional file 1: Tables S1–S3 contains tables demonstrating clinical characteristics of socioeconomic deprivation tertiles over study period. Table S1 Characteristics of socioeconomic deprivation tertile 1 over study period. Table S2 Characteristics of socioeconomic deprivation tertile 2 over study period. Table S3 Characteristics of socioeconomic deprivation tertile 3 over study period. [file 12916_2025_4137_MOESM1_ESM.docx]

**Additional file 1**

|  | **2006-08**  **(n=206)** | **2009-11**  **(n=156)** | **2012-14**  **(n=238)** | **P value** |
| --- | --- | --- | --- | --- |
| Age, *years* | 68.2 (11.4) | 71.6 (10.2) | 73.8 (11.8) | **<0.001** |
| Male, *y/n* | 160 (77.7) | 119 (76.3) | 186 (78.2) | 0.908 |
| IMD score | 8.6 (3.2) | 9.2 (3.0) | 8.7 (3.0) | 0.131 |
| BMI, *kg/m^2^* | 27.5 (5.0) | 25.2 (4.1) | - | 0.053 |
| Ischaemic aetiology, *y/n* | 135 (65.5) | 101 (64.7) | 128 (53.8) | **0.020** |
| Diabetes, *y/n* | 49 (23.8) | 37 (23.7) | 65 (27.3) | 0.618 |
| COPD, *y/n* | 15 (7.3) | 25 (16.0) | 31 (13.0) | **0.030** |
| CKD 4 or above, *y/n* | 14 (6.8) | 8 (5.1) | 22 (9.1) | 0.320 |
| NYHA Class 3/4, *y/n* | 69 (33.5) | 38 (24.4) | 54 (22.7) | **0.027** |
| LVEF, *%* | 31 (9) | 33 (8) | 31 (10) | 0.058 |
| QRS, *ms* | 124 (31) | 132 (32) | 125 (32) | 0.052 |
| SBP, *mmHg* | 120 (21) | 120 (18) | 124 (22) | 0.238 |
| DBP, *mmHg* | 71 (12) | 72 (11) | 71 (10) | 0.622 |
| HR, *bpm* | 72 (13) | 75 (16) | 75 (17) | 0.245 |
| Betablocker, *y/n* | 170 (82.5) | 132 (84.6) | 223 (93.4) | **0.001** |
| Bisoprolol equivalent dose, *mg* | 3.4 (3.0) | 4.2 (3.5) | 4.8 (3.5) | **<0.001** |
| ACEi/ARB y/n | 186 (90.3) | 146 (93.6) | 222 (93.3) | 0.394 |
| Ramipril equivalent dose, *mg* | 5.2 (3.5) | 5.3 (3.5) | 5.0 (3.6) | 0.671 |
| Loop diuretic y/n | 156 (75.7) | 112 (73.2) | 188 (77.4) | 0.707 |
| Furosemide equivalent dose, *mg* | 56 (56) | 50 (51) | 51 (45) | 0.554 |
| MRA y/n | 84 (40.8) | 60 (38.5) | 90 (37.8) | 0.805 |
| CRT y/n | 60 (29.1) | 68 (43.6) | 53 (22.3) | **<0.001** |
| ICD y/n | 38 (18.5) | 34 (21.8) | 22 (9.2) | **0.001** |

**Additional File 1: Table S1 – Characteristics of socioeconomic deprivation tertile 1 over study period.** Continuous data is presented as mean and standard deviation (SD). Categorical data is n (%). P value for continuous variables from ANOVA and categorical variables from Chi2 testing respectively. Abbreviations: angiotensin converting enzyme inhibitor (ACEi), angiotensin receptor blocker (ARB), beats per minute (BPM), body mass index (BMI), cardiac resynchronisation therapy (CRT), chronic kidney disease (CKD), chronic obstructive pulmonary disease (COPD), diastolic blood pressure (DBP), heart rate (HR), implantable cardiac defibrillator (ICD), index of Multiple Deprivation (IMD), left ventricular ejection fraction (LVEF), mineralocorticoid receptor antagonist (MRA), milligram (mg), New York Heart failure Association (NYHA), systolic blood pressure (SBP).

Footnote: Data for BMI is missing for recruitment period 2012-14.

|  | **2006-08**  **(n=219)** | **2009-11**  **(n=167)** | **2012-14**  **(n=215)** | **P value** |
| --- | --- | --- | --- | --- |
| Age, *years* | 68.2 (12.6) | 70.6 (11.9) | 72.8 (11.8) | **<0.001** |
| Male, *y/n* | 161 (73.5) | 127 (76.1) | 151 (70.2) | 0.438 |
| IMD score | 22.0 (5.5) | 22.4 (6.0) | 20.1 (5.5) | **<0.001** |
| BMI, *kg/m^2^* | 28.4 (5.3) | 28.8 (5.7) | - | 0.747 |
| Ischaemic aetiology, *y/n* | 142 (64.8) | 92 (55.1) | 123 (57.2) | 0.111 |
| Diabetes, *y/n* | 49 (22.4) | 50 (29.9) | 67 (31.2) | 0.090 |
| COPD, *y/n* | 26 (11.9) | 36 (21.6) | 38 (17.7) | **0.036** |
| CKD 4 or above, *y/n* | 18 (8.2) | 10 (5.9) | 15 (7.1) | 0.693 |
| NYHA Class 3/4, *y/n* | 75 (34.4) | 49 (29.3) | 59 (27.4) | 0.270 |
| LVEF, *%* | 32 (9) | 33 (10) | 32 (10) | 0.417 |
| QRS, *ms* | 124 (30) | 130 (33) | 122 (31) | 0.084 |
| SBP, *mmHg* | 123 (22) | 119 (21) | 127 (21) | **0.018** |
| DBP, *mmHg* | 72 (13) | 71 (10) | 71 (11) | 0.894 |
| HR, *bpm* | 73 (15) | 78 (17) | 76 (17) | 0.090 |
| Betablocker, *y/n* | 170 (78.7) | 124 (74.3) | 197 (91.6) | **<0.001** |
| Bisoprolol equivalent dose, *mg* | 3.3 (3.0) | 3.6 (3.4) | 4.1 (3.2) | **0.008** |
| ACEi/ARB y/n | 180 (83) | 146 (87) | 201 (93) | **<0.001** |
| Ramipril equivalent dose, *mg* | 4.7 (3.6) | 4.8 (3.6) | 4.9 (3.6) | 0.741 |
| Loop diuretic y/n | 171 (79.2) | 117 (70.1) | 152 (72.4) | 0.086 |
| Furosemide equivalent dose, *mg* | 54 (46) | 49 (49) | 44 (42) | **0.042** |
| MRA y/n | 89 (41.2) | 59 (35.3) | 72 (33.5) | 0.226 |
| CRT y/n | 54 (24.7) | 57 (34.1) | 43 (20.0) | **0.007** |
| ICD y/n | 29 (13.2) | 17 (10.2) | 20 (9.3) | 0.392 |

**Additional File 1: Table S2 – Characteristics of socioeconomic deprivation tertile 2 over study period.** Continuous data is presented as mean and standard deviation (SD). Categorical data is n (%). P value for continuous variables from ANOVA and categorical variables from Chi2 testing respectively. Abbreviations: angiotensin converting enzyme inhibitor (ACEi), angiotensin receptor blocker (ARB), beats per minute (BPM), body mass index (BMI), cardiac resynchronisation therapy (CRT), chronic kidney disease (CKD), chronic obstructive pulmonary disease (COPD), diastolic blood pressure (DBP), heart rate (HR), implantable cardiac defibrillator (ICD), index of Multiple Deprivation (IMD), left ventricular ejection fraction (LVEF), mineralocorticoid receptor antagonist (MRA), milligram (mg), New York Heart failure Association (NYHA), systolic blood pressure (SBP).

Footnote: Data for BMI is missing for recruitment period 2012-14.

|  | **2006-08**  **(n=196)** | **2009-11**  **(n=150)** | **2012-14**  **(n=255)** | **P value** |
| --- | --- | --- | --- | --- |
| Age, *years* | 64.8 (12.6) | 67.6 (11.8) | 68.5 (14.4) | **0.001** |
| Male, *y/n* | 136 (69.4) | 103 (68.7) | 176 (69.0) | 0.990 |
| IMD score | 48.0 (9.1) | 49.4 (10.5) | 51 (12.2) | 0.089 |
| BMI, *kg/m^2^* | 28.6 (7.4) | 28.1 (8.8) | - | 0.560 |
| Ischaemic aetiology, *y/n* | 126 (64.3) | 94 (62.7) | 126 (49.4) | **0.002** |
| Diabetes, *y/n* | 50 (25.5) | 45 (30.0) | 92 (36.1) | 0.053 |
| COPD, *y/n* | 34 (17.4) | 34 (22.7) | 45 (17.7) | 0.375 |
| CKD 4 or above, *y/n* | 16 (8.2) | 15 (10.1) | 23 (9.0) | 0.829 |
| NYHA Class 3/4, *y/n* | 85 (43.6) | 44 (29.3) | 82 (32.2) | **0.009** |
| LVEF, *%* | 31 (9) | 33 (9) | 33 (10) | **0.011** |
| QRS, *ms* | 117 (29) | 119 (29) | 118 (30) | 0.803 |
| SBP, *mmHg* | 123 (25) | 120 (20) | 125 (21) | 0.096 |
| DBP, *mmHg* | 73 (13) | 71 (11) | 71 (11) | 0.489 |
| HR, *bpm* | 74 (14) | 77 (14) | 76 (18) | 0.389 |
| Betablocker, *y/n* | 150 (76.9) | 135 (90.0) | 222 (87.1) | **0.001** |
| Bisoprolol equivalent dose, *mg* | 3.0 (2.9) | 4.5 (3.9) | 4.0 (3.4) | **<0.001** |
| ACEi/ARB y/n | 176 (90.3) | 131 (87.3) | 238 (93.3) | 0.123 |
| Ramipril equivalent dose, *mg* | 5.0 (3.5) | 5.0 (3.7) | 4.6 (3.4) | 0.330 |
| Loop diuretic y/n | 146 (74.5) | 116 (77.9) | 180 (70.6) | 0.262 |
| Furosemide equivalent dose, *mg* | 56 (57) | 50 (42) | 50 (53) | 0.332 |
| MRA y/n | 78 (40.0) | 75 (50.0) | 82 (32.2) | **0.002** |
| CRT y/n | 34 (17.4) | 41 (27.3) | 45 (17.7) | **0.033** |
| ICD y/n | 16 (8.2) | 20 (13.3) | 14 (5.5) | **0.022** |

**Additional File 1: Table S3 – Characteristics of socioeconomic deprivation tertile 3 over study period.** Continuous data is presented as mean and standard deviation (SD). Categorical data is n (%). P value for continuous variables from ANOVA and categorical variables from Chi2 testing respectively. Abbreviations: angiotensin converting enzyme inhibitor (ACEi), angiotensin receptor blocker (ARB), beats per minute (BPM), body mass index (BMI), cardiac resynchronisation therapy (CRT), chronic kidney disease (CKD), chronic obstructive pulmonary disease (COPD), diastolic blood pressure (DBP), heart rate (HR), implantable cardiac defibrillator (ICD), index of Multiple Deprivation (IMD), left ventricular ejection fraction (LVEF), mineralocorticoid receptor antagonist (MRA), milligram (mg), New York Heart failure Association (NYHA), systolic blood pressure (SBP).

Footnote: Data for BMI is missing for recruitment period 2012-14.
